# Transcanal Endoscopic Versus Microscopic Tympanoplasty: Is There a Difference in Perforation Closure Rates?

**DOI:** 10.1097/ONO.0000000000000016

**Published:** 2022-08-05

**Authors:** Tanner Mitton, Jenny Kim, Daniel E. Killeen, Jacob B. Hunter, Brandon Isaacson, Joe Walter Kutz

**Affiliations:** 1 Department of Otolaryngology – Head and Neck Surgery, University of Texas Southwestern Medical Center, Dallas, TX; 2 Department of Otolaryngology – Head and Neck Surgery, University of Alabama at Birmingham, Birmingham, AL.

**Keywords:** Tympanoplasty, Transcanal endoscopic ear surgery

## Abstract

**Objective:**

To compare closure rates of endoscopic and microscopic tympanoplasty (MT) as influenced by perforation size, perforation location, and graft position.

**Study Design:**

Retrospective chart review.

**Setting:**

Tertiary university medical center.

**Patients:**

Adult patients who underwent tympanoplasty by a fellowship-trained neurotologist from January 2010 to December 2019, had at least 2 months of follow-up, and had a tympanic perforation with no cholesteatoma before surgery.

**Interventions:**

Transcanal endoscopic tympanoplasty (ET) or MT.

**Main Outcome Measures:**

The primary outcome is postoperative closure of the tympanic membrane perforation as assessed using otomicroscopy at the last follow-up appointment.

**Results:**

Two-hundred and eleven patients—98 in the transcanal ET group and 113 in the MT group—were identified. Tympanic membrane closure rates were not significantly different between the ET and MT groups (79.6% and 84.1% respectively; *P* = 0.473), and further multivariable analysis revealed that closure rates for ET relative to MT had an insignificant odds ratio (0.56; *P* = 0.144). Similar analyses also found no significant difference between the 2 methods in subsets of perforation size (small, large, subtotal/total), perforation location (anterior, posterior, inferior), and graft position (underlay, overlay).

**Conclusions:**

ET resulted in similar rates of postoperative closure rates compared with the microscopic technique.

Transcanal endoscopic ear surgery (TEES) was first introduced in the 1990s as an alternative to microscopic tympanoplasty (MT) (1). TEES is advantageous because it provides improved visualization of the operative field, and it eliminates the need for postauricular incision, making it less invasive than MT (2). Prior studies have shown neither technique, when compared with the other, is associated with improved postoperative tympanic membrane closure rates (3), improved hearing as measured by pure tone audiometry and word recognition (4,5), or hearing outcomes in the context of ossiculoplasty (6). However, TEES requires the addition of a canalplasty less often when compared with MT (7). Two studies have compared TEES and MT in the context of anterior tympanic membrane perforations: the first analyzed 67 patients (8) and the second analyzed 100 (9). Neither study reported significant differences between rates of healing or audiometry results, though TEES was associated with less operative time. Another study compared endoscopic and microscopic approaches in overlay tympanoplasty for large perforations, finding no significant difference in hearing outcomes and graft success rates between the 2 techniques (10). Beyond these studies, no study has explored how the size and location of the tympanic perforation may warrant choosing 1 technique over the other, though at least 2 studies have included descriptive statistics of tympanic perforation location and size (11,12). The objective of this study is to compare tympanic membrane perforation closure rates between endoscopic and MT and the influence of perforation size and location as well as graft characteristics on perforation closure.

## MATERIALS AND METHODS

### Patient Selection

A retrospective chart review was conducted for patients over the age of 18 years old who underwent a tympanoplasty (CPT codes 69631, 69632, 69633) by 1 of 3 fellowship-trained neurotologists from January 1, 2010 to December 31, 2019. Each neurotologist performed both endoscopic and MT procedures, but none of them performed transcanal endoscopic tympanoplasties prior to May 2014. The approach was chosen at the discretion of the surgeon. Patients were included in the study regardless of which tympanoplasty technique they received. Patients were also included regardless of tympanic membrane surgical history, the presence of myringosclerosis, or mastoidectomy performed concurrently with the tympanoplasty. Patients were excluded if they were under 18 years of age on the day of surgery, presented with a cholesteatoma before surgery, did not have a tympanic membrane perforation before tympanoplasty, had a canal wall down mastoidectomy cavity, or had <2 months of follow-up.

### Data Collection

Demographics, characteristics of the tympanic membrane perforation, relevant medical history, surgical findings, and postoperative outcomes were collected from the electronic medical record. The size, measured as a percentage of the tympanic membrane’s surface area, and location of the tympanic perforation were determined visually by the surgeon and were recorded from the operative note; in cases where either was not included in the operative note, it was recorded from the most recent office visit before surgery. When a patient presented with multiple perforations, the characteristics of the largest perforation were recorded. Perforations were then categorized into non-mutually exclusive subsets for analysis. Perforations were categorized as large if they involved ≥50% of the tympanic membrane, small if they involved <50% of the tympanic membrane, and subtotal/total if the surgeon reported the perforation as a subtotal or total perforation in the operative note. Perforations were also categorized into anterior, inferior, and posterior subsets, according to the location of the perforation in the membrane as reported in the operative note. Perforations could be categorized into multiple location subsets if the operative note so indicated; for example, perforations that were noted as “anterior inferior” perforations would be categorized into both the anterior and inferior subsets.

Grafts were categorized into the mutually exclusive underlay or overlay subsets depending on the location of the graft relative to the anterior annulus, which was gathered from the operative note.

Data were collected and managed using REDCap electronic data capture tools (13,14). Data are available on request, with appropriate deidentification of subjects. This project was approved by our institution’s IRB (STU 012013-017).

### Data Analysis

The primary endpoint for this study was the postoperative closure of the tympanic membrane perforation. To test for significant associations between the endoscopic or microscopic technique and patient or surgical characteristics, Fisher exact test and Student *t* test were used, as appropriate. Univariate logit models with White standard errors (to control for heteroskedasticity) were then used to estimate correlations between our primary endpoint and patient or surgical characteristics. Univariate coefficients with *P* ≤ 0.20 were included in our multiple logistic regression analysis with White standard errors, as long as the variable was present in at least 10% of either the endoscopic or microscopic group (to avoid overfitting). Age, gender, and surgeon controls were always included in the multiple regression analyses, unless otherwise noted. This analysis was performed on the full dataset, as well as for each of our perforation and graft subsets (large, small, subtotal/total, anterior, inferior, posterior, underlay, overlay).

On finding a significant association between gender and closure rates, we looked for differences in the rates of all recorded variables between men and women using the same statistical tests. Variables that demonstrated different rates (defined as *P* < 0.15) between the genders were then included on multivariable analysis, along with age and surgeon controls, to try and find a cause for the gender association. A second multivariable analysis was performed to determine if overfitting of the model was the cause of the gender association. This analysis began with the univariate gender model and added in controls one by one in decreasing order of frequency in our dataset, up to 10 variables.

### Sensitivity Analysis

Given the retrospective nature of this study, there is the possibility of selection bias in our results. To address possible sources of bias, we looked at each individual factor that significantly differed between the ET and MT cohorts. We separated our patients into 2 groups for each of these factors: those that presented with that factor, and those that did not. We then performed univariate analysis of the correlation between the endoscopic technique and postoperative closure on the 2 groups separately, thereby eliminating whatever bias was contained within that factor. This process was repeated for each statistically significant difference between the ET and MT cohorts.

Statistical analyses were performed using Stata/IC 16.1 2019 (STATACorp LLC, College Station, TX). All analyses used α = 0.05 as the threshold of statistical significance.

## RESULTS

Two-hundred sixty-seven tympanoplasties were identified and 56 were excluded. This left 211 tympanoplasties for analysis: 98 in the endoscopic tympanoplasty group (ET) and 113 in the MT group. Demographics, descriptive statistics, and univariate regression results are summarized in Table 1. The tympanic membrane perforation closure rate was not significantly different between the ET (79.6%) and MT (84.1%) groups (*P* = 0.473). Large and small perforations were similarly distributed between the ET and MT groups (ET 73.5% small, 26.5% large; MT 71.7% small, 28.3% large; *P* = 0.877). There was a significant difference in the rates at which each of our 3 surgeons performed the ET and MT surgeries, with surgeon A performing 69 ET surgeries and 32 MT surgeries, surgeon B performing 17 ET surgeries and 67 MT surgeries, and surgeon C performing 12 ET surgeries and 14 MT surgeries (*P* < 0.001). There were also significant differences in the materials used for the graft in each group, as cartilage was used in 90.9% of ET surgeries but only 64.6% of MT surgeries (*P* = 0.001), while temporalis fascia was used in 4.1% of ET surgeries, but 61.1% of MT surgeries (*P* < 0.001). The transcanal approach was associated with ET surgeries (ET = 100%; MT = 5.3%; *P* < 0.001).

**TABLE 1.** Demographics and surgical characteristics

	Group comparison			Perforation closure	
	Endoscopic (n = 98)	Microscopic (n = 113)	*P* value	Univariate odds ratio	*P* value
Perforation closure	79.6%	84.1%	0.473		
Endoscopic				0.74 (0.27)	0.401
Age	47.7 (2.0)	46.4 (1.6)	0.591	1.00 (0.01)	0.909
Male	52.0%	49.6%	0.783	2.29 (0.86)	0.027^a^
Prior tympanoplasty	22.5%	36.9%	0.024^a^	0.76 (0.29)	0.468
Size			0.877		
Small	73.5%	71.7%			
Large	26.5%	28.3%		0.68 (0.26)	0.308
Location (anterior is default)			0.972		
Anterior	14.3%	11.6%	0.68		
Posterior	6.1%	4.5%	0.758	0.22 (0.19)	0.084
Inferior	23.5%	25.9%	0.75	0.96 (0.72)	0.955
Anterior superior	4.1%	2.7%	0.708		
Anterior inferior	13.3%	17.0%	0.564	0.38 (0.28)	0.184
Posterior superior	3.1%	2.7%	0.999	0.63 (0.79)	0.709
Posterior inferior	7.2%	8.9%	0.801	0.41 (0.37)	0.283
Central	28.6%	26.8%	0.877	0.53 (0.37)	0.37
Draining ear	25.5%	31.3%	0.444	1.10 (0.45)	0.819
Diabetes	9.2%	11.6%	0.655	0.96 (0.56)	0.942
Smoking history	32.7%	34.8%	0.771	1.08 (0.42)	0.846
Current smoker	11.2%	4.5%	0.074	0.61 (0.37)	0.425
Surgeon A (default)	69	32	<0.001^b^		
Surgeon B	17	67		0.80 (0.31)	0.567
Surgeon C	12	14		0.63 (0.34)	0.389
Right ear	52.0%	46.9%	0.492	0.97 (0.35)	0.923
Transcanal	100.0%	5.3%	<0.001^b^	0.75 (0.27)	0.418
Overlay graft	23.5%	29.2%	0.435	1.20 (0.50)	0.661
Lateral to malleus	7.2%	8.9%	0.801	0.48 (0.27)	0.196
Cartilage/perichondrium	90.9%	64.6%	<0.001^b^	0.86 (0.38)	0.727
Temporalis fascia	4.1%	61.1%	<0.001^b^	0.89 (0.33)	0.749
Myringosclerosis	28.6%	26.6%	0.759	2.29 (1.09)	0.081
Granulation tissue present	11.2%	18.6%	0.178	0.94 (0.47)	0.906
Infection	7.1%	13.3%	0.178	0.42 (0.21)	0.083
Canalplasty	30.6%	46.9%	0.017^a^	1.14 (0.42)	0.729
Mastoidectomy	0.0%	9.7%	0.001^c^	0.99 (0.80)	0.988

Standard error in parentheses, unless otherwise noted. Fisher exact test and the independent *t* test are used for categorical and continuous variables, respectively.

^a^*P* < 0.05.

^b^*P* < 0.001.

^c^*P* < 0.01.

The univariate logistic regression odds ratios (OR) in Table 1 demonstrate that male gender was associated with increased perforation closure rates (OR 2.29; *P* = 0.027). All other variables were insignificant on univariate analysis, though posterior perforation location was trending significant toward worse closure rates (OR 0.22; *P* = 0.084), as was preoperative middle ear infection (OR 0.42; *P* = 0.083). Preoperative myringosclerosis was trending toward improved closure rates (OR 2.29; *P* = 0.081). The results of the multiple logistic regression analysis that examines the influence of the endoscopic approach on perforation closure rates are in Table 2. The OR for the endoscopic technique was insignificant with regard to perforation closure (0.56; *P* = 0.144). Male gender was associated with improved closure rates (OR 2.46; *P* = 0.035).

**TABLE 2.** Full dataset

	Multivariable odds ratio	Confidence interval	*P* value
Endoscopic	0.56	0.26–1.22	0.144
Age	1	0.98–1.03	0.815
Gender (male)	2.46	1.07–5.67	**0.035**
Surgeon B	0.69	0.29–1.63	0.394
Surgeon C	0.57	0.19–1.74	0.322
Posterior	0.43	0.09–2.01	0.283
Anterior inferior	0.47	0.17–1.30	0.145
Myringosclerosis	2.52	0.84–7.63	0.101
Infection	0.49	0.16–1.53	0.218
R2	0.0822		

### Subsets of Patients

Table 3 contains the closure rates for each subset of patients (large, small, subtotal/total, anterior, posterior, or inferior perforations, underlay or overlay graft), as well as the OR for the endoscopic variable on multiple logistic regression analysis. For patients in the large subset, the difference in closure rates between MT (88.1%) and ET (73.5%) was large, but insignificant (*P* = 0.139). After the inclusion of control variables, the OR was trending significant to a worse closure rate with the endoscopic approach (OR 0.13; *P* = 0.060). For patients in the small subset, the closure rate in the ET group (82.5%) was similar to the MT group (83.9%; *P* > 0.999), with a multivariable OR of 0.92 (*P* = 0.863). Similarly, patients in the subtotal/total subset saw comparable closure rates with MT (83.3%) and ET (84.6%) (*P* > 0.999), and an OR of 1.79 on multivariable analysis (*P* = 0.693). Closure rates between ET and MT groups for the anterior (ET = 83.9%; MT = 82.9%; *P* > 0.999), posterior (ET = 62.5%; MT = 83.3%; *P* = 0.250), and inferior (ET = 81.4%; MT = 82.8%; *P* > 0.999) subsets of patients were similar. The OR from multivariable analyses were statistically insignificant (anterior: 2.89, *P* = 0.202; posterior: 4.02, *P* = 0.295; inferior: 0.37, *P* = 0.211). Patients in the underlay subset had higher rates of closure with the microscopic technique (86.3%) than the endoscopic technique (76.0%; *P* = 0.148). After controlling for confounding variables, the endoscopic OR was insignificant (0.63; *P* = 0.745). On the other hand, overlay patients had higher closure rates with the ET (91.3%) than MT (78.8%) surgeries (*P* > 0.282). The overlay graft subset multiple logistic regression endoscopic OR was also insignificant (0.28; *P* = 0.637).

**TABLE 3.** Subset results

Subset		Closure rates (%)					
	n	Endoscopic	Microscopic	*P* value	Multivariable odds ratio	Confidence interval	*P* value
All	211	79.6	84.1	0.473	0.56	0.26–1.22	0.144
Large	76	73.5	88.1	0.139	0.13	0.02–1.09	0.060
Small	133	82.5	82.9	0.999	0.92	0.37–2.33	0.863
Subtotal/total	37	84.6	83.3	0.999	1.79	0.10–32.08	0.693
Anterior	66	83.9	82.9	0.999	2.89	0.67–14.79	0.202
Posterior	34	62.5	83.3	0.250	4.02	0.30–54.52	0.295
Inferior	101	81.4	82.8	0.999	0.37	0.08–1.75	0.211
Underlay graft	155	76.0	86.3	0.148	0.63	0.04–10.52	0.745
Overlay graft	56	91.3	78.8	0.282	0.28	0.002–53.02	0.637

Full data on all subsets of patients, including associations between various factors and the ET and MT techniques, univariate logistic regression results, and the OR for control variables in the multiple logistic regression analyses, are included in the Supplemental Digital Content, http://links.lww.com/ONO/A4.

### Gender Association

On multivariable regression, male gender was associated with increased rates of closure in both the full dataset (OR 2.46; *P* = 0.035) and the inferior (OR 5.74; *P* = 0.004) and small (OR 3.33; *P* = 0.049) subsets. It was also trending significant in the underlay subset (OR 2.39; *P* = 0.064). In the full dataset, men were more likely to have preoperative myringitis than women (27.1% vs 16.4%; *P* = 0.068), more likely to have an anterior inferior perforation than women (19.6% vs 10.7%; *P* = 0.085) and more likely to have diabetes than women (14.2% vs 6.7%; *P* = 0.114), at our multivariable inclusion threshold of *P* < 0.15. Women were more likely to have a graft that included a porcine submucosa allograft or other non-autologous material (5.8% vs 0.0%; *P* = 0.013). The gender effect persisted on multivariable analysis with these 4 variables (OR 2.44; *P* = 0.029), and after the inclusion of age and surgeon controls (OR 2.31; *P* = 0.044). These results are in Table 4. Table 5 contains the results of the multivariable analysis that addresses possible overfitting by the incremental inclusion of the 9 variables that occurred with most frequency in our dataset into the univariate gender model and demonstrates that the gender effect persists across all models.

**TABLE 4.** Gender confounding variable exploration

	Multivariable models					
	(1)	*P* value	(2)	*P* value	(3)	*P* value
Male	2.44 (1.03–5.72)	0.041^a^	2.33 (1.04–5.24)	0.04^a^	2.31 (1.02–5.20)	**0.044**
Myringitis	0.89 (0.35–2.25)	0.81	0.86 (0.33–2.21)	0.751	0.88 (0.33–2.38)	0.807
Diabetes	0.86 (0.30–2.48)	0.782	0.83 (0.24–2.88)	0.773	0.86 (0.25–3.02)	0.815
Porcine graft	0.25 (0.05–1.30)	0.099	0.23 (0.04–1.26)	0.09	0.23 (0.04–1.28)	0.094
Anterior inferior	0.43 (0.17–1.10)	0.079	0.42 (0.16–1.08)	0.072	0.41 (0.16–1.08)	0.072
Age			1.00 (0.98–1.02)	0.923	1.00 (0.98–1.02)	0.848
Surgeon B					1.02 (0.42–2.44)	0.97
Surgeon C					0.63 (0.20–1.92)	0.413

Confidence intervals are given in parentheses. Three multivariable models are presented.

**TABLE 5.** Gender overfitting exploration

	Multivariable odds ratios									
	(1)	(2)	(3)	(4)	(5)	(6)	(7)	(8)	(9)	(10)
Male	2.29 (0.86)^a^	2.22 (0.85)^a^	2.18 (0.84)^a^	2.29 (0.91)^a^	2.28 (0.91)^a^	2.28 (0.91)^a^	2.30 (0.92)^a^	2.26 (0.89)^a^	2.35 (0.93)^a^	2.50 (1.03)^a^
Age		1.00 (0.01)	1.00 (0.01)	1.00 (0.01)	1.00 (0.01)	1.00 (0.01)	1.00 (0.01)	1.00 (0.01)	1.00 (0.01)	1.00 (0.01)
Surgeon B			0.81 (0.32)	0.77 (0.31)	0.56 (0.25)	0.56 (0.25)	0.53 (0.26)	0.53 (0.26)	0.66 (0.33)	0.71 (0.36)
Surgeon C			0.62 (0.35)	0.56 (0.32)	0.48 (0.28)	0.48 (0.28)	0.48 (0.28)	0.51 (0.31)	0.48 (0.29)	0.48 (0.30)
Size				1.00 (0.01)	0.99 (0.01)	0.99 (0.01)	0.99 (0.01)	0.99 (0.01)	0.99 (0.01)	1.00 (0.01)
Endoscopic					0.53 (0.22)	0.53 (0.22)	0.55 (0.23)	0.56 (0.23)	0.39 (0.22)	0.38 (0.22)
Right ear						0.97 (0.37)	0.96 (0.37)	0.95 (0.36)	0.99 (0.38)	0.93 (0.36)
Cartilage graft							0.82 (0.44)	0.84 (0.45)	0.69 (0.39)	0.78 (0.44)
Canalplasty								1.17 (0.49)	1.23 (0.50)	1.34 (0.57)
Temporalis fascia									0.42 (0.29)	0.43 (0.31)
Smoking history										1.30 (0.56)
Constant	3.16 (0.73)^b^	3.82 (1.80)^c^	4.32 (2.32)^c^	4.93 (2.98)^c^	8.12 (5.57)^c^	8.24 (5.80)^c^	9.74 (8.42)^c^	9.03 (8.34)^a^	15.15 (17.20)^a^	11.68 (13.08)^a^

Standard error is given in parentheses.

^a^*P* < 0.05.

^b^*P* < 0.001.

^c^*P* < 0.01.

### Selection Bias

Table 6 contains the correlations between ET and postoperative closure for each of the factors that differed between the ET and MT groups. The transcanal and mastoidectomy factors could not be analyzed in this manner because every patient in the ET group had a transcanal surgery and did not have a mastoidectomy. Among all factors that differed between the groups, there was no statistically significant OR.

**TABLE 6.** Sources of bias

	n	Endoscopic odds ratio	Confidence interval	*P* value
No prior tymp	146	0.39	0.15–1.00	0.050
Prior tymp	63	2.04	0.49–8.47	0.325
Surgeon A	101	0.68	0.20–2.31	0.535
Surgeon B	84	1.98	0.40–9.79	0.402
No cartilage	49	0.62	0.10–3.79	0.603
Cartilage	162	0.78	0.35–1.74	0.539
No fascia	138	0.67	0.24–1.82	0.430
Fascia	73	0.21	0.03–1.67	0.140
No canalplasty	128	0.4	0.15–1.04	0.060
Canalplasty	83	2.36	0.60–9.31	0.221

## DISCUSSION

Prior studies show that ET has advantages compared with MT with regards to operative time, postoperative pain, cosmetic outcomes, hospital stay, and visualization of the surgical field (15–20). Endoscopic surgery also has some disadvantages, including 1-handed manipulation of instruments, frequent cleaning of the scope, and potential for thermal injury from the endoscopic light source (21,22). In general, studies have shown that when compared with MT, ET results in comparable hearing and healing outcomes (7,20,23,24). However, the influences of perforation size, perforation location, and graft position on ET and MT outcomes have not been comprehensively studied. Given the superior visualization of the operative field with the endoscope and the easier manipulation of instruments with the microscope, it is plausible that the endoscopic approach may give better outcomes in situations where visualization is difficult, while the microscopic approach may be better for situations that require more dexterity. In other words, although each approach gives broadly similar outcomes, there may be specific instances in which one is better than the other. In this study, we retrospectively reviewed outcomes for 211 adult tympanoplasties, after excluding those with cholesteatoma, <2 months of follow-up, a canal wall down mastoidectomy cavity, and absence of perforation before surgery. Our analysis focused on perforation closure rates and included subsets based on perforation size and location and graft position, in an effort to determine if certain types of perforations may call for different instruments. No statistically significant differences in closure rates were found between the endoscopic and MT techniques in any subset, but men were found to have increased odds of perforation closure across various subsets compared with women.

Previous studies investigating outcomes of ET and MT have revealed comparable rates of healing (3–5,15,16,18,25). The present study also found no significant difference in overall closure rates, and further multivariable analysis demonstrated no difference in closure rates (OR 0.56; *P* = 0.144). Analyses were performed on subsets of perforation size (small, large, subtotal/total) and location (anterior, posterior, inferior), as well as graft position (overlay or underlay). In these subsets, no significant difference in closure rates between ET and MT were found, even after controlling for potentially confounding variables.

Within the subsets of location, anterior perforations were of particular interest, as these perforations are known to be more challenging to repair because of decreased vascularization, graft instability, and poor visualization (due to anterior bony overhang and narrow anterior tympanomeatal angle) (26–29). It is plausible that endoscopic repair could contribute to improved healing outcomes in anterior perforation repair because it allows for better visualization around the anterior bony overhang than the microscopic procedure. To date, 2 studies have compared anterior perforation outcomes between ET and MT. Both studies, Gulsen Baltaci with 67 patients and Shakya et al with 90 patients, found no significant difference in healing outcomes between the 2 techniques (8,30), a result that is consistent with our study’s analysis on anterior perforations. Our anterior perforation patient sample has nearly identical closure rates between the ET and MT groups (ET = 83.9%; MT = 82.9%; *P* > 0.999). The multivariable OR, though larger in magnitude, did not demonstrate any difference either (OR 2.89; *P* = 0.202). Interestingly, the multivariable OR for those with a canalplasty did predict improved perforation closure at a statistically significant level (OR 6.01; *P* = 0.038). We hypothesize that this may be due to improved visualization of anterior perforations following a canalplasty.

Among the subsets of size, large perforations warranted particular attention, as perforation size has been established by some studies as being a prognostic factor for tympanic membrane closure (31–34). For example, Vaidya et al (33) examined the effect of perforation size on postoperative residual perforation and found that subtotal perforations and perforations involving all 4 quadrants of the tympanic membrane were more likely to have residual perforations. Similarly, Dursun et al (34) looked at the effect of perforation size on closure rates following ET and found a negative correlation. In one study, Plodpai et al (10) compared ET and MT outcomes for overlay tympanoplasty of large perforations (defined as involving at least 3 quadrants) in 70 patients and found that healing rates were not significantly different between the 2 techniques. In our analysis of large perforations, while closure rates for ET (73.5%) appeared lower than for MT (88.1%), this did not reach statistical significance (*P* = 0.139). The multivariable OR for closure rates of ET relative to MT was trending significant (0.13; *P* = 0.060). While not statistically significant, the potential clinical consequences of a 15-percentage point difference in closure rates between ET and MT for large perforations may call for further investigation into whether there are characteristics unique to large perforations that indicate the use of MT over ET. Notably, analysis of the subtotal/total subset found no difference in closure rates between ET (84.6%) and MT (83.3%; *P* > 0.999).

The decision to use underlay or overlay graft techniques is often influenced by the type of perforation present. Underlay grafts are frequently preferred for small and easily visualized perforations, as this procedure is easier and quicker to perform and associated with faster healing time (35). However, this technique may have a higher graft failure rate in higher risk cases, such as larger or anterior perforations (31,35,36). While multiple studies have found no significant difference between ET and MT in graft uptake rates for underlay grafts (5,15,16,30,37), only 1 study to our knowledge has compared graft uptake rates for overlay grafts, also finding no difference between ET and MT (10). Our study similarly fails to reject the hypothesis that neither technique has a significant influence on healing rates in either overlay or underlay grafts.

Overall, when comparing endoscopic and MT, this study did not find any statistically significant differences when analyzing different perforation locations and sizes, as well as different graft techniques. Therefore, ET offers similar efficacy across all studied patient subsets, with less need for postauricular incision (0% ET; 94.7% MT; *P* < 0.001).

Additionally, male gender was found to be significantly associated with postoperative closure (male = 87.9%; female = 76.0%; *P* = 0.031). This gender effect persisted when controlling for possible confounding variables and with age and surgeon controls. We considered that there may be overfitting in our model due to the inclusion of excessive variables, however, the male gender OR was statistically significant in the univariate model and continued to be significant after the addition of every control variable in the multivariable model, taking on values from 2.18 to 2.50. Importantly, all variables’ OR and standard errors remained relatively constant over the 10 multivariable models. Furthermore, there was no variance in which coefficients were statistically significant. These findings suggest that overfitting is not the cause of this gender association, as overfitting would be expected to cause large changes in coefficients and standard errors from one model to the next. To our knowledge, this gender association has been found in one other study: Emir et al (38) looked at 607 patients and similarly found that male patients had a higher chance of perforation closure. However, they did not explore this finding any further.

Our analysis of possible areas of selection bias demonstrated that although the MT and ET groups were different in some ways, those characteristics did not correlate with a difference in perforation closure rates between the MT and ET groups, with one exception: in the group of patients with no history of tympanoplasty, those in the ET group were less likely to have perforation closure (OR 0.39; *P* = 0.05). The results of these analyses do not eliminate the possibility of selection bias, but they do demonstrate that the recorded imbalances between the ET and MT groups are not independently confounding the results. It is possible that other factors not directly measured are acting as confounding variables, as is the case with all retrospective studies.

### Limitations

Our study has several limitations. First, our dataset cannot differentiate between the effects of endoscopic surgery and the effects of transcanal access to the tympanic membrane. Second, the average follow-up time between the ET and MT groups was very different because no endoscopic tympanoplasties were performed until May 2014 (ET = 19.6 months; MT = 30.0 months; *P* = 0.003), so our results might change if the ET group had longer follow-up. Third, the neurotologists began implementing the endoscopic approach in the middle of the study period, so there may be a learning curve associated with ET outcomes. Finally, this study was retrospective in nature and may be prone to selection bias.

## CONCLUSION

In this retrospective review of 211 adult tympanoplasties, ET resulted in similar rates of postoperative closure compared with the microscopic technique across every subset of perforation size, perforation location, and graft position.

## ACKNOWLEDGMENTS

We thank Alexander Thomson, MSc (Columbia University) for his comments on the design and statistical methods of this study.

## FUNDING SOURCES

None declared.

## CONFLICT OF INTEREST STATEMENT

Jacob Hunter, Brandon Isaacson, and Walter Kutz are each a member of the editorial board for Otology & Neurotology Open and have been recused from reviewing or making decisions for the manuscript. No other authors have any conflicts of interest to declare.

## DATA AVAILABILITY STATEMENT

The datasets generated during and/or analyzed during the current study are not publicly available, but are available from the corresponding author on reasonable request.

## Supplementary Material


