# Supplementary material for: Transcanal Endoscopic Versus Microscopic Tympanoplasty: Is There a Difference in Perforation Closure Rates?
Source: Otol Neurotol Open. 2022 Aug 5;2(3):e016. doi: 10.1097/ONO.0000000000000016 (PMC10950148; doi:10.1097/ONO.0000000000000016)
Supplement: Supplementary file 1 [file ono-2-e016-s001.pdf]

Table A.1--Large Subset Results					
	Group Comparison			Perforation Closure	
	Endoscopic (n=34)	Microscopic (n=42)	p-value	Univariate Odds Ratio	p-value
Perforation closure	73.5%	88.1%	0.139		
Endoscopic				0.38 (0.23)	0.113
Age	47.3 (3.3)	46.7 (2.6)	0.898	1.01 (0.02)	0.464
Male	55.9%	50.0%	0.650	2.33 (1.44)	0.170
Prior tympanoplasty	17.7%	30.0%	0.281	1.09 (0.79)	0.909
Size (Median & IQR)	75% (50-90)	90% (50-90)	0.159	0.99 (0.01)	0.475
Location (Anterior is default)			0.267		
Anterior	11.8%	4.8%	0.399		
Posterior	5.9%	2.4%	0.584	0.40 (0.66)	0.580
Inferior	23.5%	16.7%	0.565	2.80 (4.25)	0.498
Anterior superior	0.0%	2.4%	0.999		
Anterior inferior	0.0%	11.9%	0.061		
Posterior superior	0.0%	0.0%			
Posterior inferior	2.9%	4.8%	0.999	0.10 (0.17)	0.164
Central	55.9%	57.1%	0.999	0.76 (0.88)	0.810
Draining ear	23.5%	36.6%	0.315	1.59 (1.14)	0.519
Diabetes	8.8%	7.3%	0.999	1.05 (1.21)	0.964
Bilateral middle ear disease	20.6%	29.3%	0.435	0.72 (0.48)	0.624
Smoking history	32.4%	39.0%	0.632	0.60 (0.37)	0.408
Current smoker	20.6%	12.2%	0.359	0.57 (0.43)	0.451
Surgeon A (default)	27	11	<0.001***		
Surgeon B	3	27		0.61 (0.40)	0.453
Surgeon C	4	4		0.25 (0.22)	0.118
Right ear	44.1%	35.7%	0.487	0.84 (0.51)	0.776
Transcanal	100.0%	0.0%	<0.001***	0.38 (0.23)	0.113
Overlay graft	44.1%	42.9%	0.999	1.48 (0.92)	0.524
Lateral to malleus	11.8%	11.9%	0.999	0.22 (0.17)	0.045
Cartilage/Perichondrium	97.1%	52.4%	0.001**	0.67 (0.48)	0.570
Temporalis fascia	5.9%	66.7%	<0.001***	0.84 (0.51)	0.776
Myringo-/Tympanosclerosis	26.5%	28.6%	0.999	0.94 (0.62)	0.931
Granulation tissue present	14.7%	23.8%	0.393	1.59 (1.32)	0.576
Infection	0.0%	14.3%	0.030*	0.41 (0.38)	0.342
Canalplasty	44.1%	57.1%	0.356	1.52 (0.91)	0.488
Mastoidectomy	0.0%	9.5%	0.123		

*p < 0.05, **p < 0.01, ***p < 0.001. Standard error in parentheses, unless otherwise noted. IQR = Interquartile range. Fisher's exact test, Mood's median test, and the independent t-test used as appropriate.

Table A.2--Large Subset Multivariable Analysis of Perforation Closure

	Multivariable Odds Ratio	p-value
Endoscopic	0.13 (0.14)	0.060
Age	1.00 (0.02)	0.829
Male	2.57 (1.91)	0.203
Surgeon B	0.19 (0.24)	0.187
Surgeon C	0.12 (0.11)	0.024*
Constant	18.6 (33.2)	0.103

*p < 0.05, **p < 0.01, ***p < 0.001. $R^2 = 0.1533$. Standard error in parentheses. Lateral to malleus variable not included due to collinearity.

Table A.3--Small Subset Results					
	Group Comparison			Perforation Closure	
	Endoscopic (n=63)	Microscopic (n=70)	p-value	Univariate Odds Ratio	p-value
Perforation closure	82.5%	82.9%	0.999		
Endoscopic				0.98 (0.45)	0.962
Age	48.3 (2.5)	45.8 (2.0)	0.415	1.00 (0.01)	0.721
Male	49.2%	48.6%	0.999	2.55 (1.26)	0.058
Prior tympanoplasty	23.8%	40.0%	0.063	0.70 (0.33)	0.447
Size (Median & IQR)	30% (15-30)	30% (20-40)	0.432	0.99 (0.02)	0.499
Location (Anterior is default)			0.856		
Anterior	15.9%	15.9%	0.999		
Posterior	4.8%	5.8%	0.999	0.14 (0.15)	0.067
Inferior	23.8%	31.9%	0.337	0.67 (0.60)	0.657
Anterior superior	6.4%	2.9%	0.424		
Anterior inferior	20.6%	20.3%	0.999	0.25 (0.21)	0.106
Posterior superior	4.8%	4.4%	0.999	0.53 (0.70)	0.629
Posterior inferior	9.5%	11.6%	0.782	0.63 (0.68)	0.668
Central	14.3%	7.3%	0.259	1.37 (1.75)	0.807
Draining ear	27.0%	28.6%	0.849	0.86 (0.43)	0.759
Diabetes	9.5%	12.9%	0.594	1.41 (1.13)	0.669
Bilateral middle ear disease	14.3%	37.1%	0.003**	1.35 (0.74)	0.586
Smoking history	33.3%	31.4%	0.854	1.90 (1.04)	0.240
Current smoker	6.4%	0.0%	0.048*	0.62 (0.73)	0.683
Surgeon A (default)	41	20	<0.001***		
Surgeon B	14	40		0.86 (0.43)	0.765
Surgeon C	8	10		0.98 (0.71)	0.978
Right ear	57.1%	54.3%	0.861	0.96 (0.44)	0.926
Transcanal	100%	8.6%	<0.001***	0.99 (0.45)	0.975
Overlay graft	12.7%	21.4%	0.251	0.99 (0.60)	0.989
Lateral to malleus	4.8%	7.3%	0.721	1.44 (1.58)	0.740
Cartilage/Perichondrium	87.3%	71.4%	0.033*	1.05 (0.59)	0.930
Temporalis fascia	3.2%	58.6%	<0.001***	0.88 (0.42)	0.783
Myringo-/Tympanosclerosis	30.2%	25.7%	0.699	4.90 (3.78)	0.039*
Granulation tissue present	9.5%	15.7%	0.312	0.64 (0.40)	0.471
Infection	11.1%	12.9%	0.796	0.40 (0.24)	0.126
Canalplasty	22.2%	40.0%	0.039*	1.07 (0.53)	0.897
Mastoidectomy	0%	10.0%	0.014*	0.50 (0.44)	0.427

*p < 0.05, **p < 0.01, ***p < 0.001. Standard error in parentheses, unless otherwise noted. IQR = Interquartile range. Fisher's exact test, Mood's median test, and the independent t-test used as appropriate.

Table A.4--Small Subset Multivariable Analysis of Perforation Closure

	Multivariable Odds Ratio	p-value
Endoscopic	0.92 (0.44)	0.863
Age	1.00 (0.01)	0.642
Male	3.33 (2.03)	0.049*
Surgeon B	1.02 (0.54)	0.967
Surgeon C	1.15 (0.89)	0.861
Anterior inferior	0.26 (0.16)	0.031*
Myringo-/Tympanosclerosis	10.40 (11.79)	0.039*
Infection	0.73 (0.49)	0.643
Constant	2.44 (1.84)	0.238

*p < 0.05, **p < 0.01, ***p < 0.001. R^2 = 0.1539. Standard error in parentheses.

Table A.5--Subtotal/Total Subset Results					
	Group Comparison			Perforation Closure	
	Endoscopic (n=13)	Microscopic (n=24)	p-value	Univariate Odds Ratio	p-value
Perforation closure	84.6%	83.3%	0.999		
Endoscopic				1.10 (1.05)	0.921
Age	50.5 (5.4)	48.7 (3.5)	0.774	1.00 (0.02)	0.866
Male	53.9%	45.8%	0.737	2.13 (2.03)	0.425
Prior tympanoplasty	30.8%	30.4%	0.999	0.61 (0.62)	0.628
Size (Median & IQR)	90% (90-100)	90% (90-90)	0.786	1.06 (0.13)	0.641
Draining ear	30.5%	43.5%	0.999	3.29 (3.92)	0.317
Diabetes	0.0%	8.7%	0.525		
Bilateral middle ear disease	23.1%	30.4%	0.716	1.64 (1.97)	0.682
Smoking history	46.2%	30.4%	0.474	0.32 (0.32)	0.253
Current smoker	23.1%	17.4%	0.686	0.96 (1.18)	0.973
Surgeon A (default)	13	6	<0.001***		
Surgeon B	0	16		0.81 (0.74)	0.820
Surgeon C	0	2			
Right ear	46.2%	37.5%	0.730	0.28 (0.26)	0.177
Transcanal	100.0%	0.0%	<0.001***	1.10 (1.05)	0.921
Overlay graft	61.5%	45.8%	0.495	1.07 (0.96)	0.943
Lateral to malleus	0.0%	16.7%	0.276	0.54 (0.68)	0.623
Cartilage/Perichondrium	100.0%	54.2%	0.003**	1.22 (1.18)	0.835
Temporalis fascia	0.0%	67.7%	<0.001***	0.72 (0.65)	0.72
Myringo-/Tympanosclerosis	30.8%	20.8%	0.691	0.58 (0.57)	0.582
Granulation tissue present	7.7%	25.0%	0.383	1.20 (1.44)	0.879
Infection	0.0%	25.0%	0.072	0.30 (0.31)	0.239
Canalplasty	61.5%	62.5%	0.999	0.79 (0.76)	0.807
Mastoidectomy	0.0%	8.3%	0.532		

*p < 0.05, **p < 0.01, ***p < 0.001. Standard error in parentheses, unless otherwise noted. IQR = Interquartile range. Fisher's exact test, Mood's median test, and the independent t-test used as appropriate.

Table A.6--Subtotal/Total Subset Multivariable Analysis of Perforation Closure

	Multivariable Odds Ratio	p-value
Endoscopic	1.79 (2.63)	0.693
Age	1.02 (0.03)	0.524
Male	1.35 (1.43)	0.776
Surgeon B	1.39 (2.11)	0.826
Surgeon C		
Right ear	0.22 (0.22)	0.129
Constant	2.69 (5.97)	0.656

*p < 0.05, **p < 0.01, ***p < 0.001. R^2 = 0.0873. Standard error in parentheses.

Table A.7--Anterior Subset Results					
	Group Comparison			Perforation Closure	
	Endoscopic (n=31)	Microscopic (n=35)	p-value	Univariate Odds Ratio	p-value
Perforation closure	83.9%	82.9%	0.999		
Endoscopic				1.08 (0.72)	0.913
Age	44.5 (3.3)	46.3 (2.8)	0.675	0.99 (0.02)	0.652
Male	54.8%	51.4%	0.810	3.71 (2.73)	0.075
Prior tympanoplasty	22.6%	37.1%	0.284	0.45 (0.31)	0.242
Size (Median & IQR)	25% (15-30)	40% (30-40)	0.014*	1.01 (0.02)	0.429
Draining ear	25.8%	14.3%	0.354	1.13 (0.97)	0.891
Diabetes	3.2%	8.6%	0.616	0.17 (0.18)	0.098
Bilateral middle ear disease	19.4%	51.4%	0.010*	1.65 (1.21)	0.499
Smoking history	16.1%	42.9%	0.031*	5.28 (5.78)	0.129
Current smoker	9.7%	5.7%	0.659		
Surgeon A (default)	23	7	<0.001***		
Surgeon B	5	27		1.08 (0.75)	0.912
Surgeon C	3	1		0.60 (0.76)	0.686
Right ear	61.3%	60.0%	0.999	0.52 (0.38)	0.377
Transcanal	100.0%	0.0%	<0.001***	1.08 (0.72)	0.913
Overlay graft	22.6%	40.0%	0.186	2.38 (1.99)	0.302
Lateral to malleus	6.7%	0.0%	0.209		
Cartilage/Perichondrium	90.3%	54.3%	0.002**	1.52 (1.07)	0.548
Temporalis fascia	3.2%	68.6%	<0.001***	0.69 (0.46)	0.575
Myringo-/Tympanosclerosis	29.0%	28.6%	0.999		
Granulation tissue present	9.7%	8.6%	0.999	0.15 (0.14)	0.039*
Infection	12.9%	8.6%	0.698	0.45 (0.41)	0.385
Canalplasty	38.7%	60.0%	0.138	5.81 (4.85)	0.035*
Mastoidectomy	0.0%	11.4%	0.116	0.58 (0.70)	0.651

*p < 0.05, **p < 0.01, ***p < 0.001. Standard error in parentheses, unless otherwise noted. IQR = Interquartile range. Fisher's exact test, Mood's median test, and the independent t-test used as appropriate.

Table A.8--Anterior Subset Multivariable Analysis of Perforation Closure

	Multivariable Odds Ratio	p-value
Endoscopic	2.89 (2.41)	0.202
Age	0.98 (0.02)	0.539
Male	2.16 (1.98)	0.401
Surgeon B	1.85 (1.65)	0.491
Surgeon C	0.91 (1.27)	0.946
Smoking history	8.21 (9.82)	0.078
Canalplasty	6.01 (5.18)	0.038*
Constant	1.17 (1.45)	0.900

*p < 0.05, **p < 0.01, ***p < 0.001. R^2 = 0.2030. Standard error in parentheses.

Table A.9--Posterior Subset Results					
	Group Comparison			Perforation Closure	
	Endoscopic (n=16)	Microscopic (n=18)	p-value	Univariate Odds Ratio	p-value
Perforation closure	62.5%	83.3%	0.25		
Endoscopic				0.33 (0.28)	0.185
Age	45.9 (4.6)	43.9 (3.5)	0.720	0.97 (0.02)	0.229
Male	43.8%	50.0%	0.744	2.17 (1.79)	0.349
Prior tympanoplasty	18.8%	16.7%	0.999	2.00 (2.38)	0.560
Size (Median & IQR)	30% (25-40)	27.5% (20-40)	0.748	0.96 (0.02)	0.096
Draining ear	18.8%	44.4%	0.152	1.97 (1.81)	0.460
Diabetes	0.0%	11.1%	0.487		
Bilateral middle ear disease	6.3%	22.2%	0.340	0.48 (0.49)	0.471
Smoking history	37.5%	27.8%	0.717	0.49 (0.40)	0.378
Current smoker	6.3%	0.0%	0.471		
Surgeon A (default)	7	7	0.743		
Surgeon B	5	8		1.33 (1.20)	0.749
Surgeon C	4	3		1.00 (1.04)	0.999
Right ear	50.0%	27.8%	0.291	1.33 (1.12)	0.729
Transcanal	100.0%	16.7%	<0.001***	0.26 (0.24)	0.143
Overlay graft	6.3%	5.6%	0.999		
Lateral to malleus	6.3%	0.0%	0.471		
Cartilage/Perichondrium	93.8%	88.9%	0.999		
Temporalis fascia	12.5%	50.0%	0.030*	1.97 (1.81)	0.460
Myringo-/Tympanosclerosis	12.5%	22.2%	0.660	0.67 (0.66)	0.680
Granulation tissue present	6.3%	27.8%	0.180		
Infection	0.0%	16.7%	0.230	0.70 (0.91)	0.782
Canalplasty	25.0%	16.7%	0.681	0.38 (0.35)	0.287
Mastoidectomy	0.0%	16.7%	0.230	0.70 (0.91)	0.782

*p < 0.05, **p < 0.01, ***p < 0.001. Standard error in parentheses, unless otherwise noted. IQR = Interquartile range. Fisher's exact test, Mood's median test, and the independent t-test used as appropriate.

Table A.10--Posterior Subset Multivariable Analysis of Perforation Closure

	Multivariable Odds Ratio	p-value
Endoscopic	4.02 (5.35)	0.295
Age	0.97 (0.03)	0.201
Male	1.28 (1.20)	0.789
Surgeon B	1.00 (1.11)	0.999
Surgeon C	1.04 (1.56)	0.979
Size	0.94 (0.03)	0.065
Transcanal	0.06 (0.10)	0.098
Constant	332.8 (965.0)	0.045*

*$p < 0.05$, **$p < 0.01$, ***$p < 0.001$. $R^2 = 0.2182$. Standard error in parentheses.

Table A.11--Inferior Subset Results					
	Group Comparison			Perforation Closure	
	Endoscopic (n=43)	Microscopic (n=58)	p-value	Univariate Odds Ratio	p-value
Perforation closure	81.4%	82.8%	0.999		
Endoscopic				0.91 (0.48)	0.860
Age	51.4 (3.0)	46.0 (2.2)	0.147	1.02 (0.01)	0.227
Male	62.8%	48.3%	0.163	3.94 (2.26)	0.017*
Prior tympanoplasty	20.9%	41.4%	0.034*	0.41 (0.22)	0.091
Size (Median & IQR)	30% (20-40)	32.5% (25-40)	0.706	1.01 (0.02)	0.726
Draining ear	23.3%	27.6%	0.653	0.88 (0.52)	0.828
Diabetes	16.3%	15.5%	0.999	1.62 (1.31)	0.549
Bilateral middle ear disease	14.0%	25.9%	0.215	2.38 (1.90)	0.278
Smoking history	32.6%	31.0%	0.999	2.69 (1.82)	0.144
Current smoker	9.3%	1.7%	0.160		
Surgeon A (default)	28	16	0.001**		
Surgeon B	9	30		0.86 (0.48)	0.789
Surgeon C	6	12		1.78 (1.51)	0.498
Right ear	51.2%	46.6%	0.691	0.93 (0.49)	0.89
Transcanal	100.0%	5.7%	<0.001***	1.06 (0.55)	0.918
Overlay graft	18.6%	19.0%	0.999	0.77 (0.49)	0.685
Lateral to malleus	4.8%	12.3%	0.294	0.70 (0.60)	0.676
Cartilage/Perichondrium	88.4%	67.2%	0.018*	1.30 (0.76)	0.661
Temporalis fascia	7.0%	60.3%	<0.001***	0.71 (0.37)	0.513
Myringo-/Tympanosclerosis	30.2%	29.3%	0.999	9.13 (9.67)	0.037*
Granulation tissue present	14.0%	17.2%	0.785	1.62 (1.31)	0.549
Infection	9.3%	10.3%	0.999	0.46 (0.35)	0.301
Canalplasty	18.6%	36.2%	0.075	0.77 (0.43)	0.635
Mastoidectomy	0.0%	12.1%	0.019*	1.32 (1.48)	0.802

*p < 0.05, **p < 0.01, ***p < 0.001. Standard error in parentheses, unless otherwise noted. IQR = Interquartile range. Fisher's exact test, Mood's median test, and the independent t-test used as appropriate.

Table A.12--Inferior Subset Multivariable Analysis of Perforation Closure		
	Multivariable Odds Ratio	p-value
Endoscopic	0.37 (0.29)	0.211
Age	1.00 (0.02)	0.846
Male	5.74 (3.50)	0.004**
Surgeon B	0.53 (0.38)	0.376
Surgeon C	1.81 (1.78)	0.545
Prior tympanoplasty	0.25 (0.18)	0.053
Smoking history	2.72 (2.47)	0.272
Myringo-/Tympanosclerosis	14.14 (18.39)	0.042*
Constant	2.86 (3.34)	0.368

*$p < 0.05$, **$p < 0.01$, ***$p < 0.001$. $R^2 = 0.2498$. Standard error in parentheses.

Table A.13--Underlay Subset Results					
	Group Comparison			Perforation Closure	
	Endoscopic (n=75)	Microscopic (n=80)	p-value	Univariate Odds Ratio	p-value
Perforation closure	76.0%	86.3%	0.148		
Endoscopic				0.50 (0.21)	0.107
Age	48.2 (2.3)	47.5 (1.9)	0.802	1.00 (0.01)	0.929
Male	45.3%	57.5%	0.149	2.38 (1.02)	0.045*
Prior tympanoplasty	22.7%	33.3%	0.154	1.21 (0.58)	0.687
Size (Median & IQR)	30% (20-50)	35% (20-50)	0.558	0.99 (0.01)	0.524
Location (Anterior is default)			0.716		
Anterior	13.3%	5.0%	0.094		
Posterior	8.0%	6.3%	0.760	0.29 (0.29)	0.214
Inferior	24.0%	28.8%	0.586	1.20 (1.08)	0.840
Anterior superior	4.0%	2.5%	0.674		
Anterior inferior	14.7%	18.8%	0.526	0.45 (0.40)	0.370
Posterior superior	4.0%	3.8%	0.999	0.83 (1.12)	0.892
Posterior inferior	8.0%	11.3%	0.592	1.08 (1.17)	0.941
Central	24.0%	23.8%	0.999	0.60 (0.52)	0.560
Draining ear	25.3%	29.1%	0.718	1.47 (0.74)	0.446
Diabetes	10.7%	13.9%	0.628	0.81 (0.49)	0.730
Bilateral middle ear disease	18.7%	29.1%	0.137	0.94 (0.46)	0.894
Smoking history	36.0%	34.2%	0.867	1.44 (0.66)	0.429
Current smoker	12.0%	2.5%	0.029*	0.35 (0.24)	0.119
Surgeon A (default)	46	30	0.006**		
Surgeon B	17	36		1.10 (0.52)	0.834
Surgeon C	12	14		0.75 (0.42)	0.608
Right ear	53.3%	45.0%	0.337	1.04 (0.43)	0.928
Transcanal	100.0%	7.5%	<0.001***	0.51 (0.22)	0.118
Lateral to malleus	9.5%	12.7%	0.612	0.49 (0.28)	0.218
Cartilage/Perichondrium	88.0%	75.0%	0.042*	0.65 (0.38)	0.456
Temporalis fascia	4.0%	52.5%	<0.001***	1.36 (0.65)	0.522
Myringo-/Tympanosclerosis	26.7%	20.0%	0.347	3.08 (1.98)	0.081
Granulation tissue present	12.0%	17.5%	0.373	1.64 (1.08)	0.455
Infection	8.0%	13.8%	0.392	0.51 (0.29)	0.239
Canalplasty	12.0%	25.0%	0.042*	1.13 (0.61)	0.823
Mastoidectomy	0.0%	11.3%	0.003**	0.79 (0.66)	0.782

*p < 0.05, **p < 0.01, ***p < 0.001. Standard error in parentheses, unless otherwise noted. IQR = Interquartile range. Fisher's exact test, Mood's median test, and the independent t-test used as appropriate.

Table A.14--Underlay Subset Multivariable Analysis of Perforation Closure

	Multivariable Odds Ratio	p-value
Endoscopic	0.63 (0.90)	0.745
Age	1.01 (0.01)	0.595
Male	2.39 (1.12)	0.064
Surgeon B	0.90 (0.47)	0.844
Surgeon C	0.70 (0.42)	0.560
Current smoker	0.46 (0.29)	0.210
Transcanal	0.79 (1.11)	0.864
Myringo-/Tympanosclerosis	4.91 (3.85)	0.042*
Constant	3.15 (2.23)	0.106

*p < 0.05, **p < 0.01, ***p < 0.001. R^2 = 0.1030. Standard error in parentheses.

Table A.15--Overlay Subset Results					
	Group Comparison			Perforation Closure	
	Endoscopic (n=23)	Microscopic (n=33)	p-value	Univariate Odds Ratio	p-value
Perforation closure	91.3%	78.8%	0.282		
Endoscopic				2.83 (2.44)	0.228
Age	46.2 (3.7)	43.8 (3.0)	0.610	0.99 (0.02)	0.715
Male	73.9%	30.3%	0.002**	2.09 (1.61)	0.341
Prior tympanoplasty	21.7%	45.5%	0.092	0.21 (0.17)	0.048*
Size (Median & IQR)	60% (30-90)	50% (30-90)	0.501	0.99 (0.01)	0.622
Location (Anterior is default)			0.952		
Anterior	17.4%	28.1%	0.522		
Posterior	0.0%	0.0%			
Inferior	21.7%	18.8%	0.999	0.83 (1.24)	0.903
Anterior superior	4.4%	3.1%	0.999		
Anterior inferior	8.7%	12.5%	0.999	0.42 (0.64)	0.566
Posterior superior	0.0%	0.0%			
Posterior inferior	4.4%	3.1%	0.999		
Central	43.5%	34.4%	0.578	0.50 (0.61)	0.572
Draining ear	26.1%	36.4%	0.563	0.53 (0.40)	0.397
Diabetes	4.4%	6.1%	0.999		
Bilateral middle ear disease	8.7%	45.5%	0.003**	1.64 (1.42)	0.569
Smoking history	21.7%	36.4%	0.376	0.48 (0.36)	0.327
Current smoker	8.7%	9.1%	0.999		
Surgeon A (default)	23	2	<0.001***		
Surgeon B	0	31		0.30 (0.26)	0.160
Surgeon C	0	0			
Right ear	47.8%	51.5%	0.999	0.77 (0.57)	0.719
Transcanal	100.0%	0.0%	<0.001***	2.83 (2.44)	0.228
Lateral to malleus	0.0%	0.0%			
Cartilage/Perichondrium	100.0%	39.4%	<0.001***	1.55 (1.15)	0.556
Temporalis fascia	4.4%	81.8%	<0.001***	0.23 (0.20)	0.089
Myringo-/Tympanosclerosis	34.8%	42.4%	0.592	1.36 (1.05)	0.693
Granulation tissue present	8.7%	21.2%	0.282	0.29 (0.25)	0.143
Infection	4.4%	12.1%	0.639	0.24 (0.24)	0.155
Canalplasty	91.3%	100.0%	0.164		
Mastoidectomy	0.0%	6.1%	0.507		

*p < 0.05, **p < 0.01, ***p < 0.001. Standard error in parentheses, unless otherwise noted. IQR = Interquartile range. Fisher's exact test, Mood's median test, and the independent t-test used as appropriate.

Table A.16--Overlay Subset Multivariable Analysis of Perforation Closure

	Multivariable Odds Ratio	p-value
Endoscopic	0.28 (0.76)	0.637
Age	1.00 (0.02)	0.984
Male	2.25 (2.24)	0.414
Temporalis fascia	0.11 (0.28)	0.392
Granulation tissue	0.47 (0.43)	0.413
Infection	0.25 (0.26)	0.191
Constant	32.61 (101.79)	0.264

*p < 0.05, **p < 0.01, ***p < 0.001. $R^2 = 0.1404$. Standard error in parentheses. Surgeon variable not included due to collinearity.
